# Long-Term Outcomes of Primary Cemented Total Hip Arthroplasty with Acetabular Bone Graft for Protrusio Acetabuli: Minimum 10-Year Follow-Up

**DOI:** 10.3390/jcm13185612

**Published:** 2024-09-21

**Authors:** Liam Z. Yapp, Nick D. Clement, Chloe E. H. Scott, Nathan Ng, Hanna P. Breusch, Deborah J. MacDonald, Paul Gaston, Steffen J. Breusch

**Affiliations:** 1 Department of Trauma & Orthopaedic Surgery, Royal Infirmary of Edinburgh, 51 Little France Crescent, Edinburgh EH16 4SY, UK; nickclement@doctors.org.uk (N.D.C.); steffen.breusch@gmail.com (S.J.B.); 2 Division of Clinical and Surgical Sciences, Department of Orthopaedics, University of Edinburgh, Chancellors Building, 49 Little France Cres, Edinburgh EH16 4SB, UK

**Keywords:** hip, protrusio acetabuli, outcome, arthroplasty

## Abstract

**Background**: This study reports the long-term survivorship of primary total hip arthroplasty (THA) for protrusio acetabuli. **Methods**: Patients undergoing THA utilising cement and bone graft acetabular reconstruction for protrusio acetabuli in a university teaching hospital during the period 2003 to 2014 were included. Kaplan–Meier survival estimates were calculated with 95% confidence intervals (CI) up to 15 years following surgery. PROMs were collected pre- and post-operatively for hip-specific function (Oxford Hip Score [OHS]) and health-related quality of life (HRQoL) using the EQ-5D-3L. **Results**: 129 consecutive THAs (96 patients) performed for protrusio acetabuli were identified (median age 69, IQR 61–75; female 115 [89.1%]; 38 [29.5%] inflammatory arthritis) with a mean follow-up of 15.7 years (range: 10.1–20.1 years). At the final follow-up, fifty-six (43.4%) patients had died and there were eleven (8.5%) reoperations, of which eight (6.2%) involved the revision of the acetabular component. The fifteen-year Kaplan–Meier any-reoperation survival estimate was 91.3% (95% CI 85.9–97.0). When considering all-cause acetabular revision only, the 15-year survival estimate was 93.1% (95% CI 88.2–98.3). The median pre-operative OHS improved significantly from baseline to 1 year post-THA, beyond the minimal important change (mean difference 28, 95% CI 25–30, *p* < 0.001). Similarly, there were clinically relevant improvements in HRQoL at 1 year post surgery (mean difference 0.10, 95% CI 0.06–0.15, *p* < 0.001). **Conclusions**: This study demonstrates that primary cemented THA utilising acetabular bone graft for reconstruction in patients with protrusio acetabuli was associated with 15-year survival rates of 93.1% and clinically relevant improvements in hip-specific function and HRQoL.

## 1. Introduction

Protrusio acetabuli is a pathological process in which the native femoral head migrates medially to the true acetabular floor, beyond the ilioischial line. Protrusio can occur primarily (idiopathic) or secondarily which is associated with several conditions including rheumatoid arthritis, ankylosing spondylitis, Pagets and following trauma [1]. In protrusio acetabuli, the severity of the deformity can progress over time, and it is associated with secondary arthritis leading to severe hip pain and disability. 

Total hip arthroplasty (THA) for end-stage arthritis is effective in reducing hip pain, improving function, enhancing quality of life [2], expectation fulfilment [3] and is a cost-effective intervention [4]. As a result, it is associated with high levels of patient satisfaction [5]. However, the majority of patients included in registry reports related to THA are broadly categorised according to the underlying disease (e.g., osteoarthritis or rheumatoid arthritis), and information regarding bony deformity is not collected [6,7,8]. 

Although the overall incidence of protrusio acetabuli is not known, it is generally considered to be a rare condition. THA for protrusio acetabuli has therefore largely been reported in small retrospective case-series with variable levels of follow-up [9,10,11,12,13,14,15,16,17,18]. The majority of long-term data either report on mixed cohorts which include revisions [19,20], the outcomes of uncemented acetabular components [13,14,16] or do not include patient-reported outcome measures (PROMs) [9,10]. 

This study aims to report the long-term clinical, radiographic and functional outcomes of the cemented and bone graft acetabular reconstruction of protrusio acetabuli in primary total hip arthroplasty (THA).

## 2. Materials and Methods

### 2.1. Study Design: Setting, Participants and Data Sources

This retrospective cohort study is reported in accordance with the strengthening the reporting of observational studies in epidemiology (STROBE) guidelines [21]. A consecutive series of patients undergoing primary cemented THA requiring bone grafting for the acetabular reconstruction of protrusio acetabuli in a single university teaching hospital during the period 1 January 2003 to 31 December 2014 were included. This study’s demographic, clinical, radiographic and PROMs data were collected after research and audit approvals were obtained (South-East Scotland (A) Research Ethics Committee 16/SS0026; Date of approval: 10 December 2020). 

Patients were identified from prospectively collected operative logbooks for two consultant orthopaedic surgeons who specialised in hip arthroplasty in our institution. Demographic and clinical data relating to the operative episode and subsequent follow-up for each patient were collected from the hospital’s electronic patient record system. Radiographic imaging including pre-operative and subsequent post-operative radiographs were reviewed using the national PACS system. Patient-reported outcome measure (PROM) scores were collected prospectively and held in the Edinburgh Orthopaedic Research Database. Subjects provided data from a questionnaire completed at the pre-operation assessment clinic and via a postal letter at the post-operative stages (12 months).

### 2.2. Operative Technique

All patients received a fully cemented THA. Surgeons used either a standard posterolateral (PG, SJB) or lateral “Hardinge” style (SJB) approach based upon their personal preference and severity of deformity. In severe protrusio cases, the posterior acetabular wall osteophyte was removed [22], and the femoral neck was cut in situ. Meticulous bone bed preparation with the drilling or burring of the commonly sclerotic roof was carried out to enhance the chance of bone graft incorporation. Acetabular floor bone grafting was utilised in all cases. The type of bone graft used depended on the size of defect. In small defects, a small slice of the femoral head was cut using oscillating saws and placed across the defect [9]. In larger defects, a combination of the patient’s native femoral head (autograft, non-lavaged) and a donated, lavaged femoral head allograft were morselised and then impacted into the acetabular floor prior to the cementing of the acetabular component [23]. An acetabular cage augment was used in cases with large uncontained defects.

The acetabular and femoral components were implanted using antibiotic-loaded cement. The acetabular components were cemented all-polyethylene components (Stryker Exeter Contemporary Flanged Cup, United Kingdom). The femoral components were both cemented and based upon the surgeon’s preference (Stryker Exeter V40 Femoral Stem, United Kingdom (*n* = 29) or a Zimmer Biomet Olympia Femoral Stem, Warsaw, Indiana, United States of America (*n* = 100)). Femoral head type selection was at the discretion of the operating surgeon.

### 2.3. Survivorship

The primary outcome of interest was overall survivorship up to 15 years post surgery. A revision procedure was considered to have taken place if the original components were surgically removed or exchanged or if new implants were added. A reoperation was any surgical procedure that was required for the left hip including revision. Stratified analyses were performed for the aseptic loosening of the acetabular component, and we included patients with clinical and radiographic evidence of aseptic acetabular loosening who were yet to be revised as events for the purpose of these analyses. Patients were censored for death, loss to follow-up or if they reached the date 30 May 2024 without experiencing a reoperation. Kaplan–Meier survival estimates were calculated with 95% confidence intervals (CI) up to 15 years following surgery. 

### 2.4. Clinical and Radiographic

Clinical data relating to immediate, early and long-term complications related to the surgery were collected retrospectively from the electronic patient record. Pre- and post-operative plain radiographs of the pelvis were scrutinised to measure the neck–shaft angle, femoral offset, acetabular offset, vertical distance and pelvic height [24]. The severity of protrusio was graded as “mild”, “moderate” or “severe” according to Sotelo-Garza and Charnley [11]. In cases of protrusio, the native hip centre of rotation (COR) is medial, and therefore the surgeon is trying to deliberately lateralise the COR to what would be “ideal” for the individual. The “ideal” or “anatomical” hip centre of rotation (COR) was calculated by determining the anatomical acetabular offset and the anatomical vertical distance using the method described by John and Fisher [25]. Restoration of the “ideal” COR was then measured by comparing the difference between the anatomical, pre- and post-operative acetabular offset and vertical height. Horizontal values could range from less than 0 (“lateralised”), 0 (“anatomic”) to greater than 0 (“medialised”). Similarly, vertical values were aiming to be around 0 (“anatomic”) with values greater than 0 (“high”) to values lower than 0 (“low”). The most recent radiographic follow-up was used to assess loosening around the cemented acetabular component [26].

### 2.5. Patient-Reported Outcome Measures

PROMs were collected for hip-specific function and health-related quality of life (HRQoL). Hip-specific function was measured using the Oxford Hip Score (OHS) [27,28]. The OHS is a validated measure of hip-specific function designed for use in patients undergoing primary THA. The OHS contains twelve questions with five possible responses, leading to a modified score which ranges from 0 (worst) to 48 (best) [27]. Clinically meaningful improvements (minimal important change (MIC)) in the OHS for patients undergoing primary THA are reported to be 11 points for the cohort and 8 points for an individual [29]. 

HRQoL was summarised using the original EuroQol 5-Dimension questionnaire (EQ-5D-3L) [30]. The EQ-5D-3L creates a health profile based upon respondents assigning three levels of impairment (“None”, “Some” and “Extreme”) to five questions related to the domains Mobility, Self-Care, Usual Activities, Pain and Discomfort and Anxiety and Depression. There are 243 potential health profiles which can be summarised as the index score, typically derived from a value set which provides preference-based weights according to the population being studied [31]. For the purposes of this study, we utilised the “Time Trade Off” value set for the United Kingdom, giving a possible numerical range from −0.594 (worst) to 1.0 (best). The MIC for the EQ-5D-3L index score in patients undergoing THA is reported to be 0.106 [32]. 

### 2.6. Statistical Analyses

Statistical analyses were undertaken using R (version 4.0.2 (22 June 2020)) in RStudio (version 1.3.959 (Boston, MA, USA)). Missing data were treated as missing completely at random. Continuous data were summarised depending on the overall distribution, with median (inter-quartile range) and mean (standard deviation) used for non-parametric and parametric data, respectively. Differences between the baseline and post-operative cohort OHS and EQ-5D-3L index scores were compared using two-sided paired Mann–Whitney U tests. Differences between categorical variables between cohorts were measured using either the Chi-squared or Fishers exact test. A *p*-value of less than 0.05 was considered statistically significant. 

## 3. Results

During the study period, 96 patients underwent 129 THAs for protrusio acetabuli (median age 69, inter-quartile range [IQR] 61–75; female 115 [89.1%]; 38 [29.5%] inflammatory arthritis) (Table 1). The mean time from surgery was 15.7 years (range: 10.0–21.0 years). The bearing selection utilised was predominantly metal (stainless steel) on polyethylene (89.9%). There were 17 cases (13.2%) that required an additional acetabular cage augment for large cavitatory defects. 

### 3.1. Survivorship and Complications

During the follow-up period, there were eleven (8.5%) reoperations, of which eight (6.2%) involved the revision of the acetabular component; fifty-six (43.4%) patients died (Table 1). The indications for reoperation were periprosthetic joint infection (*n* = 4), periprosthetic fracture (*n* = 3; open reduction internal fixation [*n* = 2], conversion to proximal femoral replacement [*n* = 1]), recurrent instability (*n* = 1), aseptic loosening of the acetabular components (*n* = 2) and soft-tissue psoas tendon release (*n* = 1). Early post-operative complications not requiring open surgical management included dislocation managed with closed reduction (*n* = 1), superficial wound infection managed with antibiotics (*n* = 2) and femoral nerve neurapraxia, confirmed on nerve conduction studies, which resolved within 1 year (*n* = 1). Post-operative medical complications included a catheter-related urinary tract infection (*n* = 1), myocardial infarction requiring angioplasty and stenting (*n* = 1), pneumonia (*n* = 1) and pseudo-obstruction of the bowel (*n* = 1). 

**Figure 1:**
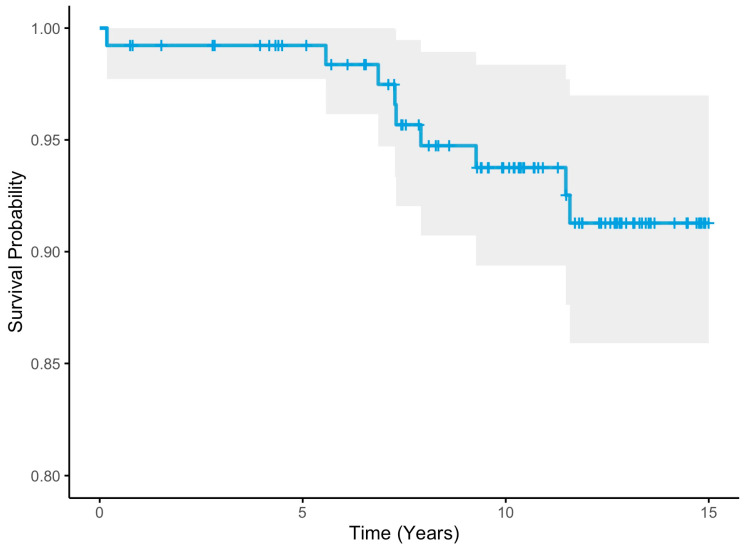
Any Reoperation 15-year Kaplan–Meier survival estimate (blue line) with 95% confidence intervals (shaded area).

**Figure 2:**
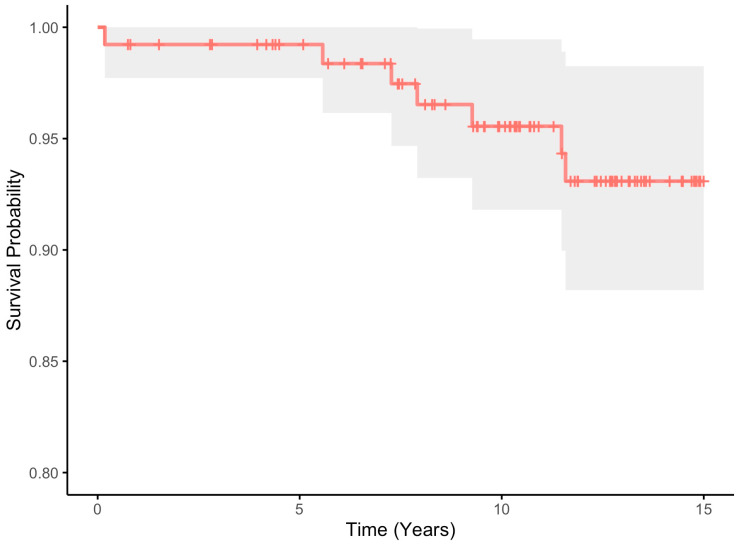
All-cause acetabular component 15-year Kaplan–Meier survival estimate (red line) with 95% confidence intervals (shaded area).

**Figure 3:**
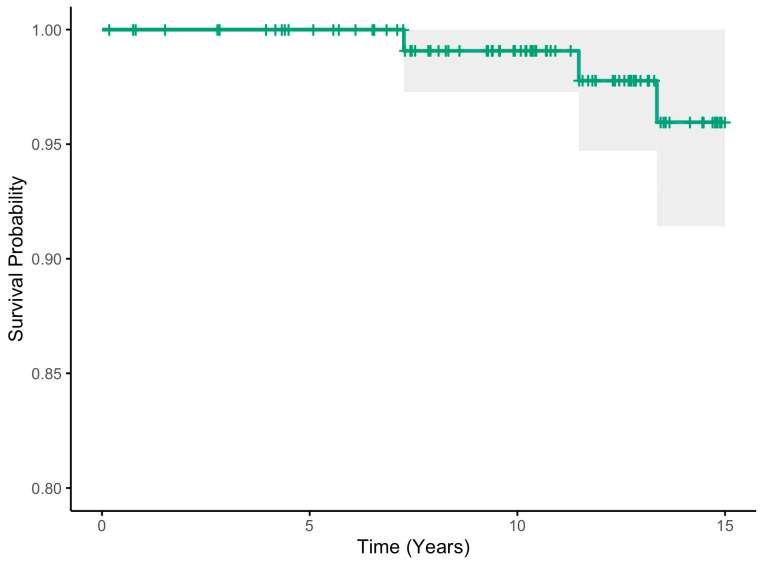
Aseptic loosening acetabular component 15-year Kaplan–Meier survival estimate (green line) with 95% confidence intervals (shaded area).

The fifteen-year Kaplan–Meier any-reoperation survival estimates for the cohort were 91.3% (95% CI 85.9–97.0) (Table 2, Figure 1). When considering acetabular revision only (septic and aseptic), the 15-year survival estimates were 93.1% (95% CI 88.2–98.3) (Table 2, Figure 2). Finally, the 15-year survival estimates for aseptic loosening were 97.8% (95% CI 94.7–100) (Table 2, Figure 3). 

### 3.2. Radiographic Assessment and Follow-Up

**Figure 4:**
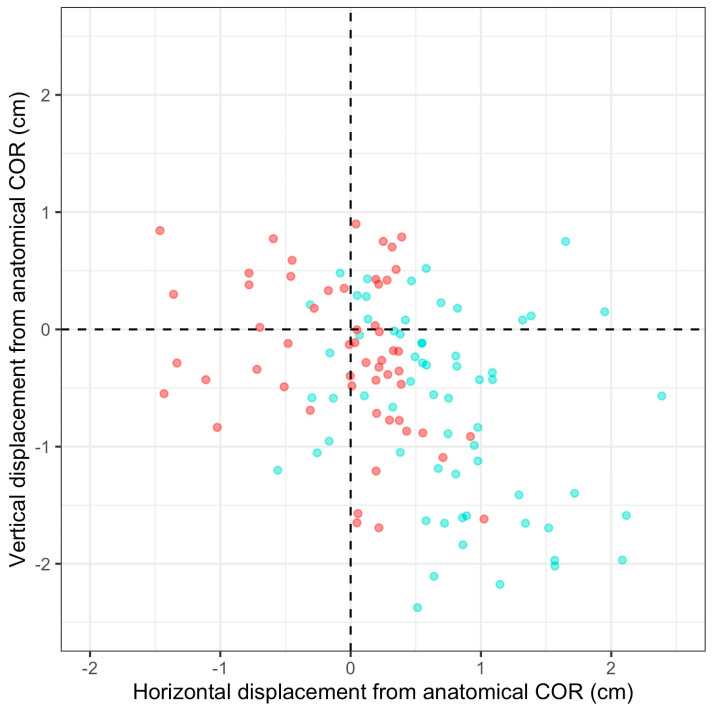
Restoration of Hip Centre of Rotation (COR). Green dots = pre-operative COR; Red dots = post-operative COR.

The majority of patients were classified as either mild or moderate protrusio acetabuli with a median distance medial to Kohler’s line of 6 mm (IQR 4–10) (Table 1). An increasing Sotello–Charnley grade of severity was significantly associated with a varus neck–shaft angle (*p* = 0.005) and reduced acetabular offset (*p* < 0.001). Following THA, the acetabular offset significantly increased and, perhaps unsurprisingly, femoral offset decreased. The difference in acetabular offset and vertical height compared to the estimated “anatomic” COR was significantly reduced (improved) following THA (Table 3) (Figure 4). 

**Figure 5:**
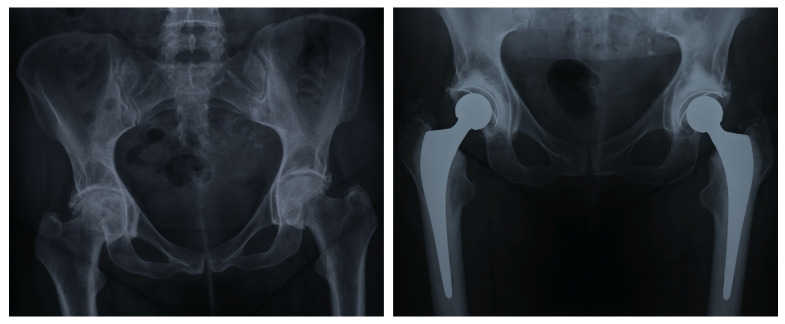
Bilateral Protrusio Acetabuli treated with fully cemented total hip arthroplasty and autogenous bone grafting (**left** to **right**: pre-operation; 2-years post total hip arthroplasty).

**Figure 6:**
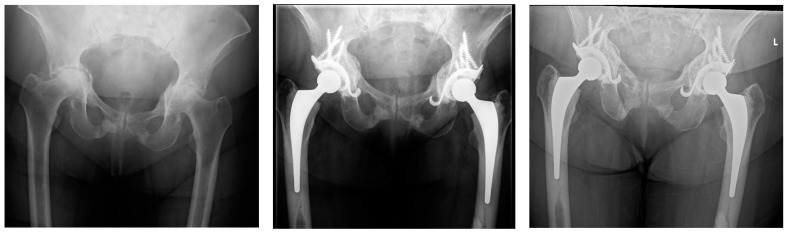
Bilateral Protrusio acetabuli treated with autogenous bone grafting, Ganz cage and cemented total hip arthroplasty (**left** to **right**): Pre-operation; 1 year post-operation; 14 years post-operation.

The median time of most recent post-operative radiographic follow-ups for the whole cohort was 10.1 years (IQR 5–13) (Figure 5 and Figure 6). The majority of patients had radiographs consistent with the incorporation of the bone graft (*n* = 127, 98.1%). There was radiographic evidence of non-progressive lucent lines (Zone I DeLee and Charnley) [26,33] without acetabular component migration in 52 THA (40.3%). Excluding cases of infection, there were four (3.1%) hips which showed radiographic evidence of acetabular component loosening and had associated symptoms.

### 3.3. PROMS: OHS and EQ-5D-3L

The median pre-operative OHS improved significantly from baseline to 1 year post-THA (pre-operation median 9, IQR 5–17) vs. post-operation median 39, IQR 31–45, *p* < 0.001). The net improvement in the OHS far exceeded the MIC (mean change 27.6, 95% CI 25–30). Similarly, the median pre-operative EQ-5D-3L index score significantly improved at 1 year post surgery (median 0.69, IQR 0.61–0.77 vs. median 0.77, IQR 0.69–0.92, *p* < 0.001). The mean change in the EQ-5D-3L was also above the threshold to be considered clinically meaningful (mean 0.14, 95% CI 0.06–0.15). 

## 4. Discussion

Primary cemented THA for protrusio acetabuli utilising bone graft for acetabulum reconstruction was associated with excellent 15-year survival. The majority of patients experienced clinically meaningful improvements in hip-specific function and HRQoL at one year post-THA. 

Pooled long-term survivorship for all primary THA from either case-series or registry reports at 15 years were reported to be 87.9% (95% CI 87.2–88.5) or 89.4% (95% CI 89.2–89.6), respectively [34]. However, more recent reports from the NJR suggest the 15-year revision risk of fully cemented primary THA to be significantly better with a cumulative incidence of revision of 5.15% (95% CI 4.99–5.31). The current literature would suggest that survival of THA in protrusio acetabuli is poorer than the average OA patient. Baghdadi et al. [15] reported a 15-year survival rate of 85% (95% CI, 68–94%) in 55 cemented THA, with no statistical difference when compared with uncemented THA. In 31 patients (36 hips) with protrusio acetabuli secondary to rheumatoid arthritis, impaction bone grafting and cemented reconstruction of the acetabulum in THA had a reported 12-year survival of 90% (95% CI 77–100) [12]. The 15-year survivorship of the current study, which reports on the outcome of 129 primary THA—the 2nd largest series reported to date—whilst being slightly better than these historic cohort studies, is in-keeping with the trend of lower overall survival when compared with the average THA patient (Table 4). In our opinion, the high rate of bone graft incorporation can be explained by meticulous bone bed preparation with the drilling or burring of the sclerotic roof and the use of non-lavaged autogenous femoral head graft and lavage for the rare cases, where additional allograft was required.

THA for protrusio acetabuli was associated with clinically meaningful improvements in the OHS at 1 year post-surgery. This finding is in-keeping with previous studies which have shown significant improvements in hip-specific function using the Harris Hip Score following THA for protrusio acetabuli [14,15,16,18,20,35]. To the authors’ knowledge, this is the first study which has also reported on HRQoL following THA for protrusio acetabuli, demonstrating clinically relevant improvements. Improvement in HRQoL is known to be associated with both the baseline and post-operative OHS score achieved in patients undergoing THA [36]. Whilst early (one-year) hip-specific function and HRQoL have been shown to improve following THA, a potential limitation of the current study was that longer-term PROM data were not collected. Whilst we cannot be sure that the observed improvements are sustained in the long-term in our cohort, emerging evidence would suggest that post-operative PROM scores remain stable up to 10 years following THA [37].

The other major limitations of this study are that it was retrospective, despite consecutive case recording and data collection by both senior surgeons, involved a single centre and did not include a suitable comparator group. Although relatively large compared to the currently available literature (Table 4), the overall sample size was small, and the relatively low numbers of events (revisions) limited the conclusions which could be drawn. Plain radiographs were not standardised for assessment, and no pre-operative templating was available for review to assess whether the planned restoration of COR had taken place. Improvement in PROM data was interpreted based upon previously published MIC for the OHS and EQ-5D-3L; however, these were generated from cohorts of predominantly OA populations, and therefore the MIC for protrusio acetabuli patients may be different.

**Table 4:** Summary of studies on THA for protrusio acetabuli survivorship since 2000.

Study	Year	N	Acetabular Fixation	Bone Graft	Follow-Up	Design	Revisions	Aseptic Loosening	Survivorship Over 10 Years
Yapp et al. *	2024	129	Cemented	129 (100%)	Mean 15.7 years	Retrospective	8 (5.4%)	5 (3.9%)	15 Year 93.1% (95% CI 88.2–98.3)
Lee BS, et al. [38]	2022	26	Uncemented	18 (69%)	Mean 5.1 years	Retrospective	0	0	N/A
Saglam et al. [39]	2016	83	Uncemented	NR	Mean 5.4 years	Retrospective	10 (12%)	3 (3.6%)	N/A
Saglam et al. [39]	2016	22	Cemented	NR	Mean 5.4 years	Retrospective	7 (31.8%)	2 (9.0%)	N/A
Baghdadi et al. [14]	2015	65	Uncemented	58 (89%)	Median 15.4 years	Retrospective	15 (23.1%)	12 (18.5%)	15 Year 85.4 (70.4–93.5)
Baghdadi et al. [15]	2013	107	Uncemented	83 (78%)	Mean 10 years	Retrospective	5 (4.7%)	3 (2.8%)	15 Year 89 (75–96)
Baghdadi et al. [15]	2013	55	Cemented	14 (25%)	Mean 10 years	Retrospective	6 (10.9%)	6 (10.9%)	15 Year 85 (68–94)
Krushell RJ et al. [16]	2008	29	Uncemented	29 (100%)	Mean 4 years	Retrospective	1 (3.4%)	0	N/A
Mullaji and Marawar [13]	2007	30	Uncemented	30 (100%)	Mean 4.2 years	Retrospective	0	0	N/A
Pereira GC et al. [20]	2007	23	Uncemented	23 (100%)	Mean 7.9 years	Retrospective	0	0	N/A
Garcia-Cimbrelo, et al. [10]	2000	148	Cemented	148 (100%)	Mean 15.7 years	Retrospective	31 (20.9%)	43 (29.1%)	NR
Rosenberg WW, et al. [12]	2000	36	Cemented	36 (100%)	Mean 12 years	Retrospective	2 (5.5%)	2 (5.5%)	12 Year 90% (77–100%)
Welten ML, et al. [35]	2000	69	Cemented	69 (100%)	Mean 12.3 years	Retrospective	4 (5.8%)	7 (10.1%)	12 Year 94%

* Current study; N = number of hips; AS = ankylosing spondylitis; RA = rheumatoid arthritis; NR = not recorded.

## 5. Conclusions

In conclusion, the long-term outcomes of the bone graft reconstruction of the acetabulum in cemented primary THA for protrusio acetabuli suggest excellent 15-year survival and clinically meaningful improvement in hip-specific function and HRQoL.

## Figures and Tables

**Table 1:** Cohort demographics.

Variable		Total, *n* (%)	Intact, *n* (%)	Revised, *n* (%)	*p*-Value
Age	Median (IQR)	70.5 (63–77)	70.0 (60.5–75.5)	65.5 (63.2–67.8)	0.224
Sex	Male	14 (10.9%)	11 (9.2%)	3 (30.0%)	0.134
	Female	115 (90.1%)	108 (90.8%)	7 (70%)	
Diagnosis	InflammatoryArthritis	31 (24.0%)	26 (21.8%)	5 (50%)	0.106
	Osteoarthritis	98 (76.0%)	93 (78.2%)	5 (50%)	
Neck–shaft angle	Median (IQR)	123 (119–128)	123 (119–128)	125 (122–128.7)	0.693
Sotello-Garza and Charnley Grade	1	54.2%	47.1%	20%	0.039
	2	39.3%	32.8%	30%	
	3	6.5%	4.2%	20%	
Mortality	Alive	73 (56.6%)	67 (56.3%)	6 (60%)	1.00
	Dead	56 (43.4%)	52 (43.7%)	4 (40%)	
Oxford Hip Score	Pre-operation, median (IQR)	9 (5–17)	9 (6–17)	6 (1–15)	0.256
	Post-operation (1 year), median (IQR)	39 (31–45)	39 (31–45)	31 (31–40)	0.242
	Change, mean (95% CI)	27.6 (25–30)	27.6	24.8	0.372
EQ-5D-3L	Pre-operation, median (IQR)	0.69 (0.61–0.77)	0.7 (0.6–0.8)	0.7 (0.6–0.7)	0.907
	Post-operation (1 year), median (IQR)	0.77 (0.69–0.92)	0.8 (0.7–0.9)	0.7 (0.7–0.8)	0.609
	Change, mean (95% CI)	0.14 (0.06–0.15)	0.1 (0.2)	0.1 (0.2)	0.678

**Table 2:** Kaplan–Meier 15-year survival: any-reoperation, all-cause revision and aseptic loosening acetabular component.

Time (Years)	Aseptic Loosening			All-Cause Acetabular Revision			Any Reoperation		
	No. Risk	Events	Survival (95% CI)	No. Risk	Events	Survival (95% CI)	No. Risk	Events	Survival (95% CI)
1	126	0	100 (100–100)	126	1	99.2 (97.7–100)	126	1	99.2 (97.7–100)
2	125	0	100 (100–100)	125	0	99.2 (97.7–100)	125	0	99.2 (97.7–100)
3	123	0	100 (100–100)	123	0	99.2 (97.7–100)	123	0	99.2 (97.7–100)
4	122	0	100 (100–100)	122	0	99.2 (97.7–100)	122	0	99.2 (97.7–100)
5	117	0	100 (100–100)	117	0	99.2 (97.7–100)	117	0	99.2 (97.7–100)
6	114	0	100 (100–100)	114	1	98.4 (96.2–100)	114	1	98.4 (96.2–100)
7	110	0	100 (100–100)	111	0	98.4 (96.2–100)	110	1	97.5 (94.7–100)
8	101	1	99.1 (97.3–100)	103	2	96.5 (93.2–99.9)	101	3	94.7 (90.7–98.9)
9	97	0	99.1 (97.3–100)	99	0	96.5 (93.2–99.9)	97	0	94.7 (90.7–98.9)
10	89	0	99.1 (97.3–100)	91	1	95.6 (91.8–99.5)	89	1	93.8 (89.4–98.4)
11	77	0	99.1 (97.3–100)	79	0	95.6 (91.8–99.5)	77	0	93.8 (89.4–98.4)
12	69	1	97.8 (94.7–100)	71	2	93.1 (88.2–98.3)	69	2	91.3 (85.9–97.0)
13	58	0	97.8 (94.7–100)	60	0	93.1 (88.2–98.3)	58	0	91.3 (85.9–97.0)
14	49	1	96.0 (91.4–100)	51	0	93.1 (88.2–98.3)	49	0	91.3 (85.9–97.0)
15	39	0	96.0 (91.4–100)	41	0	93.1 (88.2–98.3)	39	0	91.3 (85.9–97.0)

CI = Confidence Interval.

**Table 3:** Radiographic measurements—centre of rotation.

Variable		Pre-Operation	Post-Operation	Difference	*p*-Value
Acetabular Offset (cm)	Median (IQR)	2.4 (2.0–2.8)	3.3 (2.9–3.5)	−0.80 (95% CI −1.0 to −0.60)	*p* < 0.001
Vertical Height (cm)	Median (IQR)	1.9 (1.5–2.6)	1.7 (1.4–2.0)	0.29 (95% CI 0.1 to 0.5)	*p* = 0.003
Femoral Offset (cm)	Median (IQR)	4.4 (4.0–5)	4.1 (3.7–4.6)	0.36 (95% CI 0.2 to 0.6)	*p* < 0.001
Difference in Acetabular Offset From Anatomic (cm)	Median (IQR)	0.68 (0.4 to 1.1)	0.1 (−0.5 to 0.3)	0.70 (95% CI 0.50 to 0.95)	*p* < 0.001
Difference in Vertical Height from Anatomic (cm)	Median (IQR)	−0.57 (−1.4 to −0.03)	−0.26 (−0.6 to 0.4)	−0.45 (95% CI −0.76 to −0.16)	*p* = 0.002

## Data Availability

The datasets presented in this article are not readily available because of restrictions related to patient privacy. Requests to access the datasets should be directed to the corresponding author.
